# Novel methodology to examine cognitive and experiential factors in language development: combining eye-tracking and LENA technology

**DOI:** 10.3389/fpsyg.2015.01266

**Published:** 2015-08-25

**Authors:** Rosalie Odean, Alina Nazareth, Shannon M. Pruden

**Affiliations:** Project on Language and Spatial Development, Department of Psychology, Florida International University, Miami, FL, USA

**Keywords:** eye-tracking, LENA, language development, language processing, developmental systems theory

## Abstract

Developmental systems theory posits that development cannot be segmented by influences acting in isolation, but should be studied through a scientific lens that highlights the complex interactions between these forces over time (Overton, 2013a). This poses a unique challenge for developmental psychologists studying complex processes like language development. In this paper, we advocate for the combining of highly sophisticated data collection technologies in an effort to move toward a more systemic approach to studying language development. We investigate the efficiency and appropriateness of combining eye-tracking technology and the LENA (Language Environment Analysis) system, an automated language analysis tool, in an effort to explore the relation between language processing in early development, and external dynamic influences like parent and educator language input in the home and school environments. Eye-tracking allows us to study language processing via eye movement analysis; these eye movements have been linked to both conscious and unconscious cognitive processing, and thus provide one means of evaluating cognitive processes underlying language development that does not require the use of subjective parent reports or checklists. The LENA system, on the other hand, provides automated language output that describes a child’s language-rich environment. In combination, these technologies provide critical information not only about a child’s language processing abilities but also about the complexity of the child’s language environment. Thus, when used in conjunction these technologies allow researchers to explore the nature of interacting systems involved in language development.

## Introduction

Developmental systems theory posits those forces explaining child development cannot be measured as independent influences (Lerner, 2006; Gottlieb, 2007; Overton, 2013a); rather, all forces (e.g., cognitive, affective, motivational, environmental) interact to produce development over time. Attempts to determine how much of any given trait, behavior, or skill is due to one single variable will ultimately fail, as the development of these traits, behaviors, and skills are dependent on the interaction of many variables (Lerner, 2006; Partridge, 2011; Overton, 2013a). With the adoption of a developmental systems perspective to account for the development of perception, motor, and cognitive skills (e.g., Bjorklund and Brown, 1998; Spencer et al., 2009; Soska et al., 2010; Bahrick and Lickliter, 2012), developmental researchers can no longer be reductionistic; we must begin to collect data on the child’s abilities over time, while also accounting for input in the child’s environment. This will require that we not only collect data on children over time, but also collect and analyze data at different grains of analyses, and across different environments and contexts.

The study of language development is no exception; language is a hugely complex system whose development is affected by many factors, including both *cognitive factors*, such as processing efficiency, statistical learning, and phonological awareness (e.g., Stanovich et al., 1984; Fernald et al., 1998; Saffran, 2003) and *environmental factors*, like the socioeconomic status (SES) of the child’s family, the quantity and quality of language heard by the child, and the number of people with whom a child regularly spends time (e.g., Huttenlocher et al., 1991; Hart and Risley, 1995; Shneidman et al., 2009). Faced with the task of incorporating several different factors to create a comprehensive model for early language development, there is a strong need to move toward more efficient data collection methodology coupled with stronger statistical models (Urban et al., 2011). In this methodological paper, we argue for the use of combinations of sophisticated technologies to allow researchers to examine the language environment, as well as those cognitive processes influenced by and influencing language environments. Examining the dynamic interaction between the child’s language environment and their cognitive processes promotes a systemic approach to studying language development. Toward that goal, we discuss the use of two relatively new and widely accessible technology systems—eye-tracking and Language Environment Analysis (LENA) automated language processing (Xu et al., 2014) in examining language processing, where language processing is defined as how quickly and efficiently an individual attaches a word to its referent (i.e., this is sometimes referred to as real-time word recognition or comprehension; Fernald et al., 1998).

In the section on eye-tracking, we first describe traditional methods used to gather data about children’s language comprehension and processing skills and then discuss some more recent, novel approaches to analyzing language processing using eye-tracking technology. To understand language processing, eye-tracking can be used to examine how quickly children are able to map a label onto an image depicting that image. We discuss advantages in both resolution of eye movements and in differentiation of types of eye movements that are gained by using eye-tracking methodology. In the section on LENA automated language processing, we discuss what aspects of the language environment LENA can measure, its value as a tool in exploring the complex and dynamic interaction between cognitive processes and language input, and how LENA might be used to look at language input in environments other than the home setting (e.g., schools or early education settings). Finally, we examine the feasibility of combining the data-collecting capacities of these two systems to overcome the limitations of previously used methodologies with the goal of creating a holistic model of early language development. Although we restrict our discussion in the current paper to the role of parental language input on children’s language processing, we believe that the use of these technologies (along with other potential methods) jointly can and should be extended to research on other language questions of interest. Only then will we begin to understand language development from a relational developmental systems perspective (Overton, 2013b).

## Using Eye Movement to Understand Infant Language Development

Eye movement is closely linked to the human information processing system (Just and Carpenter, 1976). Eye movement as a valid and reliable measure of language processing is well documented using such methods as the intermodal preferential looking paradigm (IPLP), the looking while listening procedure (LWL), and the Visual World Paradigm with older children and adults (e.g., Golinkoff et al., 1987; Hirsh-Pasek and Golinkoff, 1996; Fernald et al., 1998; Trueswell et al., 1999; Tanenhaus et al., 2000; Trueswell and Gleitman, 2004; Pruden et al., 2006; Marchman and Fernald, 2008). Each method for gathering language data about children has its relative advantages and disadvantages. Next we discuss each method and then move to describing how eye-tracking technology can overcome some of the problems encountered in each traditional method.

### The Intermodal Preferential Looking Paradigm

In the IPLP, two images are shown on opposite sides of a split-screen during a test trial (e.g., baby; dog) and infants eye gaze to each image is videotaped. Prior to the onset of the two images, infants typically hear an auditory cue (e.g., label) asking them to attend to one of these two images (e.g., “Can you find the dog?”). Most researchers code the proportion of time infants spend looking to the correct image (e.g., dog) versus looking to the incorrect image (e.g., baby) during the test trial, with a proportion greater than 50% indicating that they have identified the correct image given the label. The assumption is that infants and children will look longer at the image that matches the auditory cue than at the image that does not match the auditory cue. This method has been widely used in the field of language development, including to study infants’ comprehension of both familiar and newly learned nouns (e.g., Golinkoff et al., 1987; Hirsh-Pasek and Golinkoff, 1996; Pruden et al., 2006), verbs (e.g., Golinkoff et al., 1987; Golinkoff and Hirsh-Pasek, 2008; Maguire et al., 2008), and other word types such as adjectives (e.g., Booth and Waxman, 2009) and spatial prepositions (e.g., Meints et al., 2002). It has even been adapted to study infants’ sensitivity to acoustic properties and phonological features of language (e.g., Swingley and Aslin, 2002; Mani and Plunkett, 2008) and children’s syntactic knowledge (e.g., Naigles, 1990; Hirsh-Pasek and Golinkoff, 1996; Lidz et al., 2003; Gertner et al., 2006). While the IPLP is a valuable tool in studying early language comprehension and children’s emerging language comprehension abilities (Golinkoff et al., 2013), it does not typically allow for the study of language processing; that is, it is not typically used to evaluate infants’ real-time word recognition or comprehension. In order for processing to be studied, eye gaze must be examined as the auditory cue is heard in “real time” (Trueswell and Gleitman, 2004). In addition, this method is prone to human error, as it requires the manual coding of infants’ eye movement to each scene or image by a human observer (though this can be done offline now). Thus, this method is not ideal for studies in which the question of interest is on real time language processing, nor is it suitable for studies involving smaller areas-of-interests within each larger image since the human coding of eye movement is limited to a comparison of course-grained, whole images.

### The Looking While Listening Procedure

More recently, Fernald et al. (1998) developed the LWL procedure to measure how quickly an infant processes a word in “real time” and shifts to look at the target image after hearing an auditory cue (e.g., label). In the LWL procedure, children view two images simultaneously on a screen (e.g., baby; dog) and then hear an auditory cue asking for the child to look to one of the two images (e.g., “Can you find the dog?”). Thus, in this procedure, children hear the auditory cue during the presentation of the video images, rather than before the presentation of the video images (as in the IPLP). The videos are then human coded frame-by-frame to determine *how quickly* infants shift to looking at the target image (i.e., dog) at the onset of the target label (i.e., “Can you find the *DOG*?”); how quickly the infant shifts to looking at the target image is thought to reflect their efficiency in processing language (Fernald et al., 2008) and serves as an index of real time language comprehension. This method has now been used to explore infants’ processing of familiar words (e.g., Fernald et al., 1998, 2010; Bergelson and Swingley, 2012), and newly learned words (Vouloumanos and Werker, 2009), as well as infants’ and children’s individual differences in language processing efficiency and their later language, cognitive, and academic abilities (Fernald et al., 2006; Marchman and Fernald, 2008; Marchman et al., 2010). Critically, use of the LWL method has also allowed for the exploration between language input and children’s language processing, finding that those children who hear more language in the home are more efficient (“quicker”) to process words (Hurtado et al., 2008, 2014). Like the IPLP, the LWL procedure is limited in the resolution with which children’s visual gaze can be analyzed; human coding of eye movement for LWL is limited to comparison of course-grained, whole images and is still subject to human error. In addition, a unique problem to the LWL procedure is that in cases where the infant was already attending to the target image at the onset of the target word, no data on the child’s efficiency in processing language is gathered, resulting in missing data (in some cases, half of the trials will have missing data) and less power to detect significant differences. Unfortunately, the LWL procedure, well suited for gathering data on infants’ language processing, still suffers from the very same spatial resolution problems as the IPLP allowing for only course-grained analyses of images, and like the IPLP, is more susceptible to human coding error.

### Eye-tracking Technology to Examine Infant Language Processing

The use of eye-tracking technology is a fairly recent methodological tool used to evaluate infants’ language processing efficiency or real-time language comprehension (also see the *Visual World Paradigm* with older children and adults to study real time language processing; e.g., Tanenhaus et al., 1995). Research in infant language processing can greatly benefit from the development of eye-tracking technology in that it can: (1) allow for the presentation and analysis of complex visual displays; (2) provide detailed temporal information; (3) reduce the likelihood of human coding error, as well as decrease time for manual coding; and (4) allow for talk across technologies.

#### Eye-tracking in Split Screen Paradigms

First, eye-trackers allow researchers to examine how infants comprehend, as well as process, real-time language in a way that has not previously been possible, through complex visual displays and detailed temporal information. For example, a manual coding approach, such as those used in the former IPLP (Hirsh-Pasek and Golinkoff, 1996) and LWL paradigms (Fernald et al., 1998), is the gold standard in calculating visual attention in children and typically allows the researcher to gather information about the length of time to an image (i.e., a fixation). However, eye-tracking technology has made it possible to inspect additional types of eye movements (e.g., fixations *and* saccades), often with little to no human error, thus expanding on the scope of the research questions that can be studied through eye-movement. By looking *only at fixations*, rather than all eye gaze data (i.e., fixations *and* saccades), researchers can focus on the time during eye gaze when linguistic information is being actively processed (as reflected by fixations), but will lose information about the time during which attention is shifting (as reflected by saccades). Hand coding is limited to the resolution with which visual attention can be coded, with most studies focused on looks to a target image, to a distractor image, between the images, or away from the screen (e.g., Fernald et al., 1998).

Eye-tracking, on the other hand, allows areas of interest (AOI) to be defined in specific regions of the screen, such as a part of an image (e.g., Huettig and Altmann, 2005). Use of this high-resolution eye gaze allows for within-group comparisons, including the comparison of looking patterns across children who performed similarly on a language-processing task. There may be differences in how infants process visual information, which could be reflected in what parts of an image they fixate on and how they move between images. Eye-tracking allows researchers to look at visual attention within 5° of eye movement, rather than limiting them to the mere up, down, left and right eye movements of children. This means that we can learn not just what image an infant is fixating on, but on what part of the image, which may be reflective of what information is being processed at a given time. This also allows for designs where a researcher is interested in looking at eye gaze to more than two predetermined locations on a screen. For example, in a study with adults, Huettig and Altmann (2005) used an eye-tracker to examine looking patterns to displays of four images. Three types of displays were run, ones containing the target image, ones containing a conceptual competitor (e.g., a conceptual competitor for *rabbit* was *pig*), and one containing both the target on competitor. For each trial, the remaining spaces on the screen were filled with unrelated images, such that four images were always shown. By using an eye tracker, the researchers were able to examine the proportion of fixations to a specific image (i.e., target, competitor, or distractor), over time. The study found that during the target condition, more saccades were directed toward the target than distractors. In the competitor condition, there were more saccades toward the competitor than distractor. In the target and competitor condition, the most saccades were toward the target, but more saccades were toward the competitor than the distractor. These findings, showing how visual gaze relates to semantic processing, would have been exceedingly difficult to uncover without the use of an eye tracker.

In addition to allowing for more complex stimuli to be used, eye-tracking also permits researchers to answer complex “temporal questions” about language processing. To some degree this has been possible with previous approaches, such as the LWL procedure, with researchers coding shifts in visual attention just prior to and after onset of a target noun. Nation et al. (2003) looked at how language processing differed between children with high and low reading comprehension. Participants 10–11 years old participated in measures of reading and phonemic decoding, as well as an eye-tracking measure. The eye-tracking measure looked at children’s ability to use verbs that restrict possible nouns to shift to an image of a noun. The sentences included phrases like, “Jane watched her mother *eat* a cake,” in which cake was the only food image shown, versus, a sentence where the noun was not predictable from the verb, “Jane watched her mother *choose* a cake.” In addition, filler sentences were included where the noun was either near the beginning or middle of the phrase. Four images were displayed on a screen in quadrants. Children were told to touch an image when they heard its name. Trials where the child selected the wrong image were excluded from analysis. Eye-tracking was used to explore anticipatory looks between the verb onset and the noun onset. Eye-tracking measures found that children were able to use the supportive verbs (e.g., *eat*) to anticipate the noun, with significantly more anticipatory looks to the target image in the supportive verb condition. Furthermore, children with lower reading comprehension scores, showed more anticipatory looks overall, across conditions. This study again shows the utility of eye-tracking technology when exploring eye gaze to quadrants, rather than the left–right split screen used in many preferential looking studies (e.g., Fernald et al., 1998; Pruden et al., 2006), as well as in exploring more complex temporal questions by examining anticipatory looks.

#### Technological Advantages of Eye-tracking

By eliminating the need for human coders, eye-tracking greatly reduces the element of human error while simultaneously decreasing the time needed for manual coding. Eye-tracking technology is equipped to differentiate between fixations and saccades. Fixations are pauses in eye movements over a specific visual AOI, while saccades are rapid eye movements between fixations (Salvucci and Goldberg, 2000). They are an important source of information often difficult (to near impossible to code) and very time-consuming to measure when manually coding eye movement. For example, the Visual World Paradigm, primarily used to examine adult processing of sentences in real-time, has been adapted for use with children as young as 3-years-old (e.g., Tanenhaus et al., 1995; Pyykkönen et al., 2010). Like the LWL approach, the Visual World Paradigm uses eye gaze to determine real-time processing of words (Huettig et al., 2011). However, rather than relying on extensive hand coding of eye-gaze, the Visual World Paradigm utilizes eye-tracking technology to determine how sentences are processed (Tanenhaus et al., 1995). Similarly, Trueswell et al. (1999) used a head-mounted eye-tracker to explore how 4- and 5-year-old children processed sentences in real time. Children viewed a live display with objects in four quadrants (e.g., a frog toy, a frog on a napkin, a napkin, and a box, each in their own quadrant) and heard a request (e.g., “Put the frog on the napkin in the box”). Some of the requests were ambiguous, as with the previous example, where you either can put the frog and napkin in the box, or put the frog found on the napkin in the box. This study found that, like the adults who had previously been studied, 5-year-olds show incremental processing of sentences in real time. Though similar looking-time studies have previously been conducted using hand-coding (e.g., Fernald et al., 1998; Marchman and Fernald, 2008), by using eye-tracking technology this study was able to accurately detect when the participant shifted to each of four images.

#### Integrating Eye-tracking with Other Technology

Laboratory set-ups that allow for the simultaneous recording of the stimuli seen by the child and the child’s eye gaze have previously involved many components (e.g., a projector screen, video cameras, multiple computers, a time code generator, and in some cases outdated equipment including VCRs; Fernald et al., 2008). Eye-tracking combines these technologies into a single user-friendly system, allowing eye gaze data and video stimuli to be synced in real-time. Eye-tracking methodology has proved not only useful for data collection purposes, but also for data analysis functions. The built-in fixation filter algorithms available on most eye-tracking systems parse eye movement data into saccades and fixations, which can then be used in conjunction with open-source programming software like Python, as well as purchasable software like MATLAB, to create gaze-data plots for further analysis.

The value of eye-tracking technology as a reliable and valid measure of language processing suitable to study children’s language processing abilities (Tanenhaus et al., 2000) cannot be undermined, however, we do caution that eye-tracking technology may not necessarily serve as a complete replacement for human coders. The reliability of eye-tracking data may diminish if infants move their heads, which can make the eye-tracker temporarily lose track of the infants’ eyes (Oakes, 2012). Further, eye-tracking data is only as accurate as the calibration done at the beginning of a study, during which an infant watches a small animation in the corners and center of the screen, and the eye-tracker records where the infant’s eyes are fixated (Oakes, 2012). All subsequent eye-tracking data depend on the accuracy of this calibration (Oakes, 2012). Because of the difficulty in calibrating eye trackers with infants, calibration beyond that built into eye-tracking software is recommended (Frank et al., 2012). These approaches usually involve having infants watch a second set of calibration points following the automated calibration, to see if accurate data is being collected (Frank et al., 2012). Although eye-tracking has generally very high temporal and spatial resolution, there may be some latency in measurement, especially when using eye-tracking with multiple softwares (e.g., e-prime or Matlab; Morgante et al., 2012). These latencies are especially concerning for tests of processing speed where small differences may affect whether an eye movement is classified as related to an audio cue or not. Researchers should keep this in mind, and test their own set-up, even if it meets or exceeds the requirements suggested by the manufacturer (Morgante et al., 2012).

### Using Eye-tracking to Understand Spatial Language Processing: A Test Case

**FIGURE 1:**
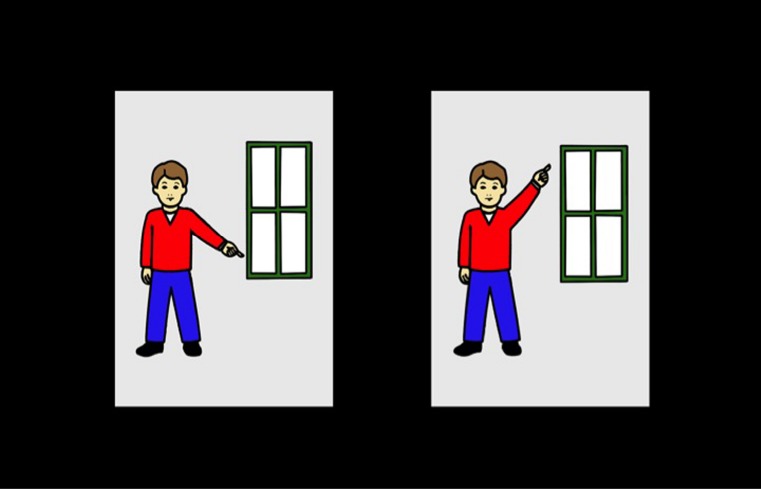
**A test trial from our processing efficiency of familiar spatial terms study.** While viewing the following image a child would hear, “*Can you find the boy pointing to the bottom of the window*?”

We currently use eye-tracking technology to explore individual differences in toddlers’ comprehension and processing of spatial language. Spatial language refers to terms used to describe location, direction, shape, dimension, features, orientation, and quantity of objects (e.g., *near*, *left*, *rectangle*, *large*, *corner*, *forward*, *same*). Using the Tobii X60 eye tracker, we examine how quickly 3-year-olds shift to an image depicting a target spatial relation in real time upon hearing a target spatial word. For example, in a typical test trial, children see two corresponding spatial scenes on opposite sides of a screen (Figure 1; e.g., images of a boy pointing to the top of a window and of a boy pointing to the bottom of a window). Their visual attention is recorded as they hear an auditory cue asking them to look to one of the images (e.g., “Can you find the boy pointing to the *bottom* of the window?”). This allows us to look at comprehension in a number of ways. To determine if a child comprehends a term, we look to see if the child spends more time across the trial looking to the target image (e.g., boy pointing to bottom of window) than to the distractor image (e.g., boy pointing to top of window). By looking at the latency of the first fixation to the target after the onset of the target term (i.e., “bottom”) we can explore how efficiently a child can map a familiar spatial term onto its corresponding image. Finally, by looking at the specific fixations, we can explore what part of the image children use to identify the spatial relation (e.g., children might look at the pointing arm to determine if the boy is pointing up or down); note, this is not something that can be done using traditional methods like IPLP or LWL. Previous studies have used eye-tracking to look at children’s processing of sentences in real-time, and how they move from one image to another (e.g., Trueswell et al., 1999). By looking at children’s visual attention to specific areas within images, we might be able to better understand children’s processing of the complexities of spatial language. For example, when asked to find the boy pointing to the bottom of the window, do children spend more time looking at the edges of the window or do they follow the arm of the boy? Looking at these data may allow us to further understand individual differences in processing speed by not only examining how quickly children shift attention, but also exploring what feature or object within an image draws their attention during different parts of a test trial. While the overall measure of comprehension could have been obtained with manual coding, only eye-tracking allows the spatial resolution and temporal evidence to compare what parts of the image children look at as they hear and process each part of the auditory cue.

In sum, eye-tracking technology can be used to examine the language processing abilities of infants and toddlers, and there are several new advantages to using eye-tracking technology over traditional language processing procedures. The biggest advantage of eye-tracking over previous approaches such as the IPLP or LWL paradigm is the increased spatial and temporal resolution obtained from eye-trackers. Eye-tracking allows researchers to look at attention to many images on a screen simultaneously, or to specific regions within an image. In addition, eye gaze can be explored during specific parts of a trial, or the order of fixations in time can be explored. This increased spatial and temporal resolution has helped to broaden the types of research questions that can be studied. Furthermore, eye-tracking reduces human error and decreases the coding time associated with manual coding. While eye-tracking allows for researchers to answer many questions about children’s real-time language processing abilities, we are also interested in exploring the dynamic naturalistic language environment as a predictor of language processing abilities in children.

## Studying the Language Environment Using LENA

Studies looking at children’s language environment have been an important part of the study of language development for many decades, and have contributed extensively to our understanding of how language emerges within a naturalistic context (e.g., Brown and Bellugi, 1964; Hart and Risley, 1995). These studies rely on recording samples of children’s spontaneous speech within their homes, schools, or other environments where the child typically spends their time. Speech samples are then transcribed and coded using established systems such as CHAT (MacWhinney, 2000), SALT (Systematic Analysis of Language Transcripts; Miller and Chapman, 2000) or some other valid/reliable system. Studies have looked at children’s mean length of utterance (MLU; the average number of morphemes per utterance), the quantity of words used, the number of unique words used, as well as the language heard by the child (e.g., Brown and Bellugi, 1964; Hart and Risley, 1995; Hoff, 2003; Huttenlocher et al., 1991; Pruden et al., 2011). Methods for collecting samples of language have relied primarily on audio and/or video recordings, along with traditional transcription systems, or on diary studies in which parents or caregivers report when their child has produced specific words or utterances. Below we describe each of these traditional methods for collecting language samples and then discuss a modern system for studying a child’s language environment, the LENA system (Xu et al., 2014).

### Audio and/or Video Recording and Traditional Transcription of Child Language

In an extensive study of two children’s language development, Brown and Bellugi (1964) conducted one of the first systematic investigations chronicling the emergence of syntax. One child, Adam, was followed beginning at age 27 months, while the other, Eve was followed from age 18 months. Each child’s spontaneous production of speech was examined through 2 h tape recordings made in their homes every 2 weeks for a year. In addition to the collection of tape recordings, a researcher made a transcript of speech and events during the home visits. This study looked primarily at the length and types of utterances children used, as they moved from utterances averaging less than two morphemes in length (Adam averaged 1.84, and Eve 1.40 at the start of the study), to utterances over three morphemes in length (3.55 and 3.27 by week 38, respectively). In addition to following the development of utterance length, Brown and Belligu examined the types of word combinations children used (e.g., “determiner + noun” or “noun + verb”). This now classic study laid the groundwork for later language studies by using a systematic approach to naturalistic language assessment. This classic study of children’s language production, however, utilized a small sample size likely because these longitudinal speech samples required time-intensive transcription and coding. This problem is not one that is unique to Brown and Belligu’s early study but is seen in many studies of children’s language development.

For example, groundbreaking research by Hart and Risley in the 1990’s (1995) explored the effects of family SES on children’s language environments, and in turn, on children’s language development. Using audio-recordings, this study explored the language experiences of children from 42 families of diverse SES, with 13 professional, 23 working-class, and 6 welfare families participating in the study. Families were observed for 1 h a month for every month when the child was between 10- and 36-months-old. Not only did parents from higher SES families use more words overall with their children than families of lower SES, they also used more complex and diverse speech. Both vocabulary growth and vocabulary use at age 3 years was predicted by family SES. Twenty-nine of the children from the original sample participated in a follow-up study of academic performance at age 9–10 years (Walker et al., 1994; Hart and Risley, 1995). Rate of vocabulary growth at age 3 and language use at age 3 predicted language scores on multiple measures, including the Peabody Picture Vocabulary Test—Revised (PPVT-R) and the Test of Language Development (TOLD) at age 9–10. In addition, language use at age 3 predicted reading comprehension skill at 9–10. These lasting effects point to the importance of the early language environment not only in early language development, but also for later academic performance. Though the sample in this study, was much larger than Brown and Bellugi (1964), and looked at a more diverse sample of families, language was only recorded for 1 h at each month. This hour may not be reflective of the overall experience of that child, as children may have different caregivers at different times of day or different days of the week. Further, this method required the time-intensive transcription and coding of language samples.

The prevalence of the study of language through naturalistic transcripts becomes obvious when one first sees the CHILDES database (MacWhinney, 2000). Thousands of articles have been published using the CHILDES database, begun at Concordia University in 1984 as a database of freely available transcripts of children’s speech (e.g., Rescorla et al., 2001; Huttenlocher et al., 2002; Perfors et al., 2011). The transcripts in this project have provided data to address many questions in language development, but many questions still require researchers to collect original language samples by recording and transcribing the language of children. Each transcript represents hours of work beyond the original recording time. Because of this researchers may choose not to use naturalistic language samples in a study, or may use transcripts representing only a small fraction of a child’s day (e.g., 1 or 2 h). New technology, which we will review shortly, can overcome some of these challenges.

### Diary Studies of Child Language

To study the emergence of specific language skills (e.g., when particular words are produced for the first time) researchers have used diary studies of child language, in which every instance of a certain word type or utterance is recorded, often by the parent or caregiver (Tomasello, 1992; Naigles et al., 2009). Unlike transcriptions, which provide a detailed snapshot of the child’s language use, a diary study looks at the development of usages of particular words (or utterances) by recording in a diary when and where that word (or utterance) was produced. The diary study approach was integral to early language development research, as it allowed for the naturalistic study of language without the use of recording devices (e.g., Stern and Stern, 1928). Even with advances in technology, this approach is still well-suited to aspects of language that might rapidly change, where their development might be missed using samples every month, or even every 2 weeks, as is common in transcription studies.

Tomasello (1992) used the diary study method to examine the development of first verbs using a single child’s (his daughter) language production from 12- to 24-months. From 12- to 17-months, Tomasello and his wife recorded each non-nominal (i.e., not a label) expression and all word combinations, along with the context in which these expressions and utterances took place. For early uses, they also attempted to record adult-child interaction that gave rise to the expression or utterance. Between 17- to 20-months, the diary method was combined with audio- and/or video-recordings of semi-naturalistic dyadic interactions, between the child and her mother and the child and her father, recorded at the beginning of the month lasting 30 min each. From the 20th month, common verb-noun combinations were no longer recorded as Tomasello became interested in the child’s more sophisticated and mature uses of language. This study provides a clear view of how one child transitioned from her immature uses of verbs and the contexts in which they took place to mature uses of verbs, as well as sophisticated verb combinations later in the second year. Groundbreaking in its comprehensive coverage of verb uses, this study was limited in sample size with only the one child.

To understand trajectories in verb production (and other types of words), larger samples are necessary. Recently, efforts have been made to capture verb use in a larger sample utilizing the diary method. Naigles et al. (2009) had eight mothers record their children’s first 10 uses of 34 verbs thought to be amongst those first used by children. Results suggest that children used verbs to both command and describe, to talk about a wide variety of actors and objects, and across a variety of different syntactic structures. While this study represents a substantially larger sample than previous language diary studies, and uses a clear systematic approach that could be implemented by multiple families in the same way, the sample is still smaller than is typical of even language studies using audio/video recordings of language. Finally, by only asking mothers to record the use of 34 verbs, other early verb uses and structures may have been missed. Many of the limitations encountered in both traditional audio/video recording and transcript studies, as well as in diary studies, can effectively be dealt with by using automated language analyses, such as the LENA system (Xu et al., 2014).

### LENA Technology to Examine A Child’s Language Environment

The LENA system was developed to provide researchers with an automated language analysis method to collecting large-scale recordings of naturalistic home environments. Thus, this system allows the researcher to track and examine individual differences in language development trajectories across large samples of children (Xu et al., 2014). The LENA system can record for up to 16 continuous hours and the Digital Language Processor (DLP) device which records the audio is small enough to be worn by an infant or young child in a special shirt or vest. To date, the LENA system has primarily been used to study children with atypical language development (e.g., Caskey et al., 2011; Irvin et al., 2012), though see recent efforts to use the system with typically developing children to track and increase the amount of language children hear from their parents in the home setting (Suskind et al., 2013). We propose that the LENA system be used as a measure of typical children’s language development, alongside traditional measures of receptive and expressive vocabulary, in order to gain a more complete picture of the child’s skill level, the child’s language environment, and the role of adult language input in predicting the child’s skill level. The LENA system has many advantages including: (1) increased ecological validity with less or no experimenter influence on recorded interactions, as well as increased sampling of language data; (2) the ability to quickly and automatically identify language- and/or conversational-rich areas of the recording for further transcription; (3) the capability to provide timely feedback on caregiver language in intervention studies; and (4) the means to examine the link between typically developing children’s language skills and their language environment.

#### Using LENA as an Alternative to Transcription

By utilizing the LENA system researchers have available to them thorough and automated information about children’s language environments for entire days, providing increased ecological validity over previous approaches. The LENA system allows researchers to record long naturalistic language samples, and provides automatic estimations of the number of child vocalizations (in older children we would call these words), adult words, and conversational turns between a child and adult(s) during the recording (Caskey and Vohr, 2013). While these automated LENA data calculate only the quantity of language in the environment, these data are a very powerful tool for exploring children’s spontaneous speech environment without the interference of a researcher during recordings, and without the necessity of time-intensive transcription and coding (though transcription is required if the researcher wishes to look at specific words or word types).

In addition to using the automated output to examine the quantity of language children use and hear, the LENA output can be used to identify specific areas in the audio file for further transcription and examination. This helps address the problem in previous transcription studies where it is hard to tell if a sample is representative of the child’s typical language environment. With the automated LENA output, a typical sample can be selected within a recording (e.g., first continuous 20 min segment of the recording), a random sample can be selected within a recording (e.g., a random selection of continuous 20 min segments regardless of time of day), or a sample can be selected by another quantitative measure (e.g., richest continuous 20 min of child vocalizations).

#### LENA in Intervention Studies

Language Environment Analysis can also be used in intervention studies, where the need to give timely feedback can make transcription an impractical tool. Suskind et al. (2013) used the LENA system to explore the success of an intervention to increase the amount of language used by 17 non-parent caregivers of typically developing 10- to 40-month-olds. Recordings were taken at eight time points, including two baseline measures taken prior to the intervention, and six post-intervention recordings. The intervention consisted of a look at the child’s language development, as well as tips for increasing conversational turns and overall speech quantity. Caregivers were provided with the LENA output from their two baseline recordings, including raw numbers, percentiles, and graphs of adult word count and conversational turns between child and caregiver. Adult word counts from post-intervention recordings were significantly higher than baseline measures, though the rate of conversational turns and the number of child vocalizations did not increase. This study did not look at children’s language outcomes, but points to the utility of the LENA system as a tool for collecting language samples from typically-developing children and their caregivers, as well as the potential of using the system in interventions to increase the amount of language a child hears.

#### LENA and Typical Language Trajectories

Use of the LENA system allows for the automated examination of the relation between typically-developing children’s language skills and their language environment (Weisleder and Fernald, 2013; Ramírez-Esparza et al., 2014). Weisleder and Fernald (2013) recently published data using the LENA system to explore the relation between the infant’s language environment and infants’ language processing efficiency for object labels. Twenty-nine lower-SES Spanish-speaking 19- to 24-month-old infants participated in a two-part study. Families were recorded when the infant was 19 months using the LENA system in the home setting. LENA recordings from participating families ranged from 3 to 13 h. Human coders transcribed 60 min of recording from 10 of the 29 recordings, with a high correlation between the human coder estimates of adult word count and the LENA system estimates, confirming the accuracy of the LENA system for use with Spanish-speaking adults. Coders also listened to 5-min segments of the recordings to determine if speech was primarily child-directed or overheard providing a measure of how much child-directed speech was heard at 19 months. Children participated in a looking-while-listening task, a lab-based measure of language-processing efficiency for familiar nouns, at both 19 and 24 months. At 24 months, caregivers also completed the Spanish-language version of the MacArthur-Bates Communicative Development Inventories, the MacArthur-Bates Inventario del Desarrollo de Habilidades Comunicativas: Palabras y Enunciados (Jackson-Maldonado et al., 2003). This parent report provided a measure of the child’s expressive vocabulary size. A significant relation was found between the amount of child-directed speech heard at 19 months, and language processing efficiency at both 19 and 24 months, which held even when controlled for child vocabulary at 24-months. A mediation analysis suggests that processing efficiency partially mediates the relation between the amount of child-directed speech heard at 19 months and vocabulary size at 24 months. This study points to the utility of LENA output as a measure of children’s overall language input and their language environment. Using the LENA system to measure language input and traditional methods, such as LWL to measure language processing efficiency, we can efficiently explore those important interactions between the language environment and language development.

Although the LENA system is a powerful tool, users must keep in mind a few of the limitations. The algorithms are normed based on 12-h recordings, during which the device was worn by a small child (Xu et al., 2014), and thus may not result in accurate output for shorter samples or when worn by adults. Additionally, the LENA system can only tell us about the quantity of language used; transcription is still necessary for questions regarding the types of language used.

#### Using LENA to Examine the Child’s “Spatial” Language Environment: A Test Case

Current research in our lab uses the LENA system to explore the role of the language environment in children’s development of spatial reasoning and the development of spatial language. We are using the LENA system to explore the language environment in pre-kindergarten classrooms, and the extent to which educator language in these classrooms influences children’s growth in early spatial and numeracy skills. Recordings must still be transcribed in order to examine specific types of language used (e.g., coding for spatial words and coding for talk about numbers), but LENA not only makes the transcription process easier by allowing us to hone in on language-rich segments, it also provides automated language data about individual speakers. LENA algorithms divide recordings into speaker segments based on the frequency and decibel level of the audio signal; differences in frequency and decibel level distinguish different speakers and the algorithm then assigns different codes for a female adult, target child, male adult, and non-target children. This facilitates the transcription process by helping a transcriber identify who is speaking, and move more quickly through a transcription. In addition, LENA provides data on the average decibel level and peak decibel level in a classroom, factors that also potentially contribute to the quality of a child’s learning environment and subsequently, their academic outcomes. Finally, the LENA DLPs can be used to explore individual differences in the amount of language heard by children both within the same classroom and between classrooms and to estimate how early educators divide their time amongst their students. Taken together, these advantages suggest that the LENA system is a valuable tool for descriptive, experimental, and intervention studies. Its utility as a measure of language experience and language production for studies in formal and informal learning environments as well as home environments makes it a powerful resource for all language researchers.

## Joint Use of Eye-tracking and LENA Technologies to Examine Language Development

As we have just seen, both eye-tracking technology and the LENA system are valuable tools for examining children’s language development. Eye-tracking technology is particularly useful in gathering data on children’s online processing of words in real time, yielding a language processing efficiency measure for individual children. The LENA system is a valid and efficient tool for examining the language input children hear from caregivers in their home and school environments. While there is no denying the usefulness of each technology in isolation, there is a lot to be gained by combining the data-collection capacities of both technologies.

Using human coders for manually calculating eye gaze frame by frame, and for language transcription and coding, is a time-intensive, expensive process. By using eye-tracking technology and software, and automated language processing software available through LENA, researchers can collect the same reliable and valid data and produce automated output quickly. These automated methods can also finally allow for the collection of larger sample sizes that had been previously prohibitive with traditional looking time and language sampling methods. It is also worthwhile to note that with rapid advancements in eye-tracking hardware, researchers now have access to head mounted eye-trackers that can be used to study visual exploration experiences of infants as young as 13-months (Franchak et al., 2011; Smith et al., 2014). Advanced head-mounted eye-trackers offer the researcher the luxury of obtaining eye gaze data under more naturalistic conditions, such as when children are interacting with objects and people in their environment. Head mounted eye-trackers in combination with automated language recorders like the LENA system, have made the once time-intensive, error-prone naturalistic observations of children less prohibitive to the developmental scientist. Further, rapid improvements in eye-tracking software has led to the development of newer features like dynamic AOIs, which allow for the use of dynamic stimuli like events, dynamic movies, and even social interactions.

Preferential looking procedures have often been used in early language research, both to examine infants’ comprehension of familiar words (e.g., Fernald et al., 1998) and to look at novel word learning (Pruden et al., 2006; Pruden et al., in preparation). Although most of these studies have relied on human coders to evaluate infant looking patterns, eye-tracking can be utilized in these studies to get a more nuanced view of children’s visual attention, including the calculation of how “efficient” the child is at processing words in “real-time.” These calculations of processing efficiency reflect individual differences in the child’s language ability. Individual differences in performance on these language tasks is often predicted by infants’ prior language ability and often predicts how large the child’s vocabulary will be at a later age (Fernald and Marchman, 2012).

Work by Hart and Risley (1995) as well as others (Huttenlocher et al., 1991; Hoff, 2003), has shown the importance of the early language environment on children’s language development. Traditional methods for gathering naturalistic language samples in the home setting require time-intensive transcription and coding. By including the LENA system in language studies, researchers are able to connect the effects of the language environment and children’s language processing in a way that has not previously been possible (Weisleder and Fernald, 2013). Though other measures can estimate how many words children know, by recording up to 16 h of a child’s natural language environment, LENA provides a unique opportunity to understand how much children talk and are talked to. In conjunction with eye-tracking data looking children’s real-time language processing, LENA output can provide a more complete picture of the factors that influence the child’s language development, including their language environment. Thus, jointly, these two methodologies begin to provide a picture of the whole child’s language abilities and how these language abilities develop.

In our own experience, we have found both the LENA system as well as the Tobii X60 eye tracker to be efficient and reliable in collecting data on children’s language environment and their own language production as well as children’s real-time language processing and comprehension. We are now interested in combining these methodologies in new studies to establish a more comprehensive approach to examining early language development. With this goal in mind, we have designed a two-part language study that investigates those cognitive and environmental factors that may predict and explain children’s language development.

In the cognitive component of this project, we examine children’s ability to comprehend and process familiar spatial terms using eye-tracking. In this lab-based experiment, a child views images on a split-screen depicting two different spatial concepts (Figure 1; e.g., boy pointing to bottom of window; boy pointing to top of window). While viewing these two images, the child is asked to look to one of these images using a short phrase with a spatial term (e.g., “can you find the boy pointing to the bottom of the window?”). Eye-tracking allows us to measure how quickly children process familiar spatial terms resulting in the calculation of the child’s processing efficiency for spatial words. We can calculate how quickly the child shifts to the correct image upon hearing the target spatial word (e.g., bottom). Critically, and something unique to the use of the eye-tracker, we can examine the specific spatial location (AOI) children look to within the images upon hearing the target spatial term. This is a feature unique to eye-tracking and one that cannot be obtained with traditional methods like LWL.

In the environmental component of this project, we are interested in examining the role of parental language input and its association with performance in the lab-based language processing efficiency eye-tracking study. We expect that children who hear more spatial language at home, and who have more conversational turns about spatial activities with their caregiver will be faster at processing familiar spatial terms, have a more complete understanding of familiar spatial terms, and potentially be more efficient at learning novel spatial terms. In this naturalistic experiment study, we are visiting the child’s home environment to record language use via the LENA system during dyadic interactions between caregiver and child. This allows us to get an accurate understanding of whether the quantity and quality of home language plays a similar role in the processing of spatial terms to what has been seen with the processing of nouns in Fernald’s work (Hurtado et al., 2008, 2014). Thus, in a single study we are examining how a child’s environment, and early spatial language input, as well as the child’s real-time processing efficiency of familiar spatial terms interact in the development of spatial language.

## Conclusion

A developmental systems perspective emphasizes the importance of interactive systems to understanding human development, including the development of language (Lerner, 2006; Lerner and Benson, 2013; Overton, 2013a). Without exploring development within the context of the environment we cannot fully understand the individual differences we see throughout development. Technology to examine language development in the young child has certainly come a long way since the first reported diary studies, however, we are not yet using modern-day technologies to their fullest capabilities to address the rapidly evolving approach to studying language development. In this paper, we argue for the interactive capabilities of eye-trackers and the LENA system. Technologies like eye-tracking and the LENA system, when used in conjunction, allow researchers to collect data that encapsulates the complexities of interacting systems in development. As part of the research community, the sooner we adopt these technologies and use them in novel combinations, the faster will there be a push for the development of software scripts (e.g., MATLAB, PYTHON, R-scripts) that allow these powerful technologies to “talk” to each other in ways suited to our field. As technology shifts at a rapid rate, it is a great time for researchers to open dialogue with software and hardware developers to collaboratively develop research-specific features in these new technologies. The versatility of these technologies to be used in laboratory and naturalistic environments offsets the initial investment of capital and human effort to a large extent. We hope that these major advantages will inspire researchers to investigate the potential uses of these exciting technologies for their own language and cognitive development research questions.

### Conflict of Interest Statement

The authors declare that the research was conducted in the absence of any commercial or financial relationships that could be construed as a potential conflict of interest.
